# Account of a Remarkable Case of Vaccination

**Published:** 1812-11

**Authors:** 


*To the Editors of the Medical and Physical Journal.*

GENTLEMEN,

A REMARKABLE Case of Vaccination occurred in my district in March last, the symptoms of which, as they appeared, I will enumerate; and, should it be worthy the insertion in your useful vehicle of theoretical and experimental knowledge, be pleased to insert it.

On the 12th of March last, I was desired to vaccinate a boy of four years of age, belonging to H. Carr. I did not see him till the eighth day after vaccination, when no appearance of inflammation was present; concluded that the vaccination had not been effectual; resolved on a second trial, which was performed on the other arm; on the sixth day the punctured part began to put on the appearance of a regular pock; and on the eighth day I charged six lancets, and was on the point of taking my leave, when the mother of the boy said, "dear me, sir, there is another pock on the arm you first inoculated," and to my surprise there really was. On my calling about three days after, I found it was much enlarged. Both the margin and pustule bore the physiognomy of a pustule after vaccination, and the cicatrix was perceptible in the middle where the arm was punctured, but did not seem to have arrived at maturity. I called again in two days, and found it still more enlarged, and took some fluid on purpose to try whether it would affect another child if vaccinated with it, or not, and accordingly put it in execution. The child on whom the experiment was tried, was four years of age. It made the usual appearance on the fourth day, and seemed a promising pock, and on the ninth day it changed its appearance to a bluish yellow, and terminated in the usual manner. The margin and pustule of this pock were remarkably large, larger larger than any I ever saw, but that might probably arise from habit.

What induced me to transmit to you this extraordinary case, is to know from what cause both arms took the infection, and at such distant periods; what was the cause of the quiescence of the first inoculation; and on what principle the second child took the infection from a pock that was so long in coming to maturity? Admitting it was at maturity at the time, how could it be possible that from its age it would affect another child, and that child's pock come to maturity, and end in the usual manner. I hope that some of the learned contributors to your Journal, will give me some reply as to probability, or whether it is a singular phenomenon, or whether such a case ever came under their investigation or not. The possibility of such a case may be disputed, but it is ready to be attested immediately, should any doubt take place.

I am, Gentlemen,

